# Epidemiological characteristics and genetic alterations in adult diffuse glioma in East Asian populations

**DOI:** 10.20892/j.issn.2095-3941.2022.0418

**Published:** 2022-11-01

**Authors:** Zongchao Mo, Junyi Xin, Ruichao Chai, Peter Y.M. Woo, Danny T.M. Chan, Jiguang Wang

**Affiliations:** 1 Division of Life Science and State Key Laboratory of Molecular Neuroscience, The Hong Kong University of Science and Technology, Clear Water Bay, Kowloon, Hong Kong SAR, China; 2 HKUST Shenzhen-Hong Kong Collaborative Innovation Research Institute, Shenzhen 518000, China; 3 Department of Molecular Neuropathology, Beijing Neurosurgical Institute, Capital Medical University, Beijing 100070, China; 4 Department of Neurosurgery, Kwong Wah Hospital, Hong Kong SAR, China; 5 Hong Kong Neuro-Oncology Society, Hong Kong SAR, China; 6 Division of Neurosurgery, Department of Surgery, Prince of Wales Hospital, Hong Kong SAR, China; 7 Department of Chemical and Biological Engineering, The Hong Kong University of Science and Technology, Clear Water Bay, Kowloon, Hong Kong SAR, China; 8 Hong Kong Center for Neurodegenerative Diseases, Hong Kong Science Park, Hong Kong SAR, China

**Keywords:** Glioma, East Asian, epidemiology, germline, somatic

## Abstract

Understanding the racial specificities of diseases—such as adult diffuse glioma, the most common primary malignant tumor of the central nervous system—is a critical step toward precision medicine. Here, we comprehensively review studies of gliomas in East Asian populations and other ancestry groups to clarify the racial differences in terms of epidemiology and genomic characteristics. Overall, we observed a lower glioma incidence in East Asians than in Whites; notably, patients with glioblastoma had significantly younger ages of onset and longer overall survival than the Whites. Multiple genome-wide association studies of various cohorts have revealed single nucleotide polymorphisms associated with overall and subtype-specific glioma susceptibility. Notably, only 3 risk loci—5p15.33, 11q23.3, and 20q13.33—were shared between patients with East Asian and White ancestry, whereas other loci predominated only in particular populations. For instance, risk loci 12p11.23, 15q15-21.1, and 19p13.12 were reported in East Asians, whereas risk loci 8q24.21, 1p31.3, and 1q32.1 were reported in studies in White patients. Although the somatic mutational profiles of gliomas between East Asians and non-East Asians were broadly consistent, a lower incidence of *EGFR* amplification in glioblastoma and a higher incidence of 1p19q-*IDH*-*TERT* triple-negative low-grade glioma were observed in East Asian cohorts. By summarizing large-scale disease surveillance, germline, and somatic genomic studies, this review reveals the unique characteristics of adult diffuse glioma among East Asians, to guide clinical management and policy design focused on patients with East Asian ancestry.

## Introduction

Gliomas account for more than 80% of all primary malignant tumors affecting the central nervous system (CNS)^1^. According to the 2007 World Health Organization (WHO) classification of CNS tumors, the diagnosis of gliomas was predominantly based on histological hallmark features including glioblastoma (GBM), diffuse astrocytoma, anaplastic astrocytoma, oligodendroglioma, and anaplastic oligodendroglioma^2^. In the 2016 version, isocitrate dehydrogenase (*IDH*) mutation and 1p/19q codeletion status of these tumors were also considered crucial biomarkers for integrated diagnosis^3^. According to the most recent 2021 version, adult diffuse glioma now includes 3 molecular types: GBM, *IDH*-wild-type; astrocytoma, *IDH*-mutant; and oligodendroglioma, *IDH*-mutant and 1p/19q-codeleted^4^. Given that molecular information is frequently lacking in the published literature, this review broadly classified gliomas into 3 histological types: GBM, astrocytoma, and oligodendroglial tumors.

The etiology of glioma remains unclear. Both environmental and genetic factors may increase the risk of this disease^5–7^. Exposure to ionizing radiation at younger than older ages is associated with a relatively higher risk of developing glioma^6^. A recent genome-wide association study (GWAS), a common approach used for genotype-phenotype association discovery, has estimated the heritability of glioma to be 6.69%, thus implying the existence of genetic variants that contribute to the heritable risk of glioma^8,9^. Other GWAS studies have identified several single nucleotide polymorphisms (SNPs) associated with the risk of diffuse gliomas^10–12^. Because cancer is caused by the accumulation of somatic mutations in hallmark genes, large sequencing projects such as The Cancer Genome Atlas (TCGA) have investigated genome-wide data from hundreds of patients with glioma and reported somatic mutations in *IDH1*, *TP53*, *PTEN*, *EGFR*, *NF1*, and other genes^13–15^. These sequencing studies have enhanced the current understanding of gliomagenesis and provided potentially actionable targets for precision oncological management.

**Figure 1:**
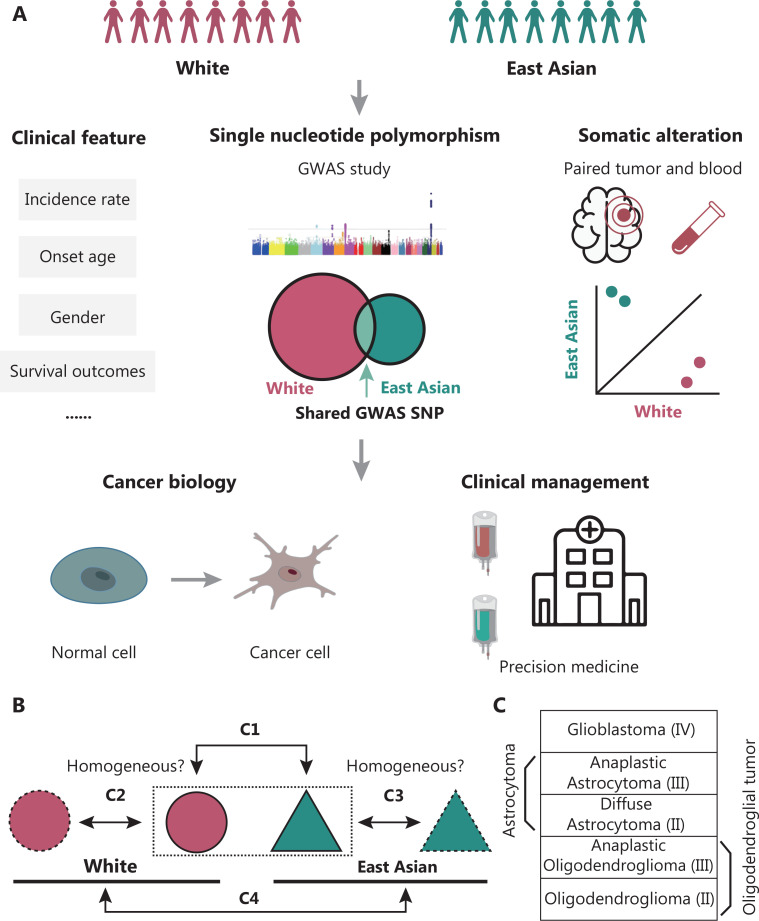
Schematic workflow of this review. (A) By focusing on East Asian and White populations, we first reviewed clinical features, including incidence rate, median onset age, gender, and survival differences for adult diffuse glioma. Second, we retrieved GWAS research and reported risk SNPs for both populations. The larger circle in the Venn plot represents a larger enrolled White population. Third, we summarized somatic landscape differences between the ancestry groups. Finally, we discussed the potential significance of ancestry differences in understanding glioma cancer biology and personalized treatment. (B) First (C1, the prefix C indicates comparison), to address differences between the populations from a racial/genetic perspective, regardless of environmental factors, we compared people of East Asian *vs.* White descent from the same country (mainly the USA or UK). The dashed-line rectangle indicates the same country/environment. Second (C2), we compared independent studies in people of White ancestry from different countries to evaluate the homogeneity among Whites. Third (C3), we retrieved studies based on East Asian countries or regions, such as China, Japan, and South Korea, and estimated the homogeneity among East Asians, which was further compared with that in people of East Asian ancestry in the USA or UK, to assess the consistency within East Asian ancestry. Finally (C4), after homogeneity estimation, we compared the East Asian and White ancestry. Owing to the lack of data or the organization of the framework, the sequence of C1 to C4 for specific sections could not always be strictly followed. (C) Subtypes included for comparison. The WHO grade is also shown. Astrocytoma included both diffuse astrocytoma and anaplastic astrocytoma. Oligodendroglial tumors included both oligodendroglioma and anaplastic oligodendroglioma.

Recently, large-scale epidemiological studies in patients from multiple racial groups have demonstrated interesting patterns of racial differences in adult diffuse gliomas^16,17^. Independent studies from different geographical regions have also indicated distinct epidemiological and genetic characteristics^18–24^. This review focuses on the genetic factors contributing to the differences in glioma specific to East Asian patients. In particular, we concentrated on glioma studies from Chinese, Japanese, and Korean populations, and compared their glioma incidence, survival outcomes, genetic alterations, and other clinical factors to those in White populations [non-Hispanic White (NHW)/White] (**Figure 1A**).

The observed differences among these patient cohorts might have arisen from non-genetic factors, such as the procedures used for diagnosis, statistical methods, and potential regional environmental risk factors. To mitigate these confounding effects, we assessed data from different populations exposed to similar environmental factors, such as those from the same country, for control comparisons^16,17^. We also compared East Asian patients from China, Japan, and South Korea with Whites from western countries (**Figure 1B and 1C**). A comprehensive review of epidemiological characteristics, glioma susceptibility variants, and somatic mutation profiles was performed (**Supplementary Table S1**).

## Epidemiology characterization

### Incidence of gliomas

Recent studies have implied that glioma incidence is partially associated with race. Using a UK database, Maile et al.^17^ have found that, the incidence of gliomas among patients of South Asian or Chinese ancestry was significantly lower (*P* < 0.01) than that in White patients; the corresponding relative incidence rate ratios (IRR) were 0.58 and 0.68, respectively. In the USA, the age-standardized incidence rate (ASR, per 100,000 per year) among White patients (ASR = 6.45) was higher than that among Asian and Pacific Islander (API) patients (ASR = 3.20)^25^. Independently, the ASR of all gliomas was approximately 6, and showed no significant change from 2000 to 2016^26^, whereas the glioma ASR among White patients was 6.22^27^. In contrast, the ASR of high-grade gliomas in China was 1–4^28^. The overall glioma ASRs were 2.76 and 2.89 in Kumamoto and Miyazaki prefectures respectively^29,30^. The Korean population also showed a similar ASR of 2.82^31^. Overall, the ASR among White patients was approximately 2 times higher than that in East Asians. In the following, we discuss the incidence of each diffuse glioma subtype.

#### GBM

**Figure 2:**
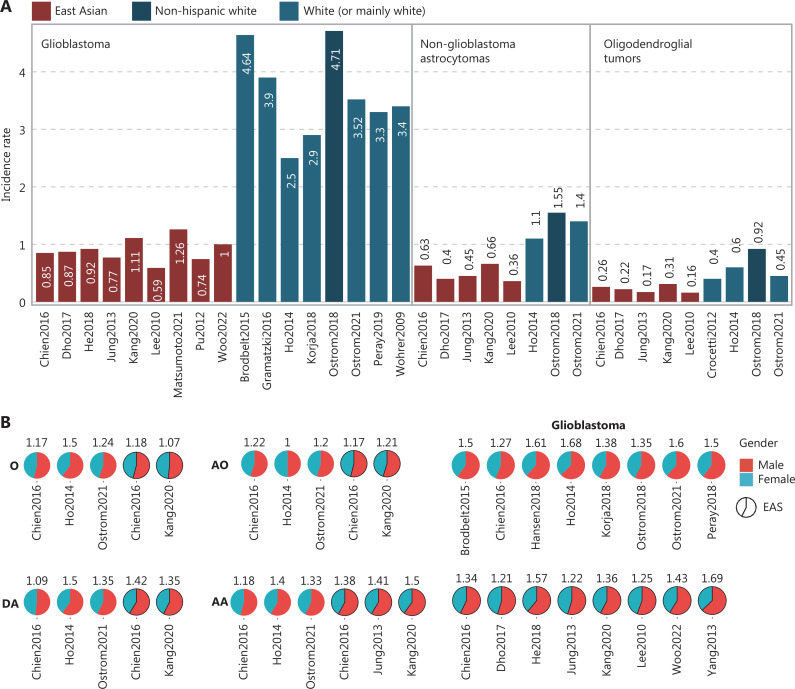

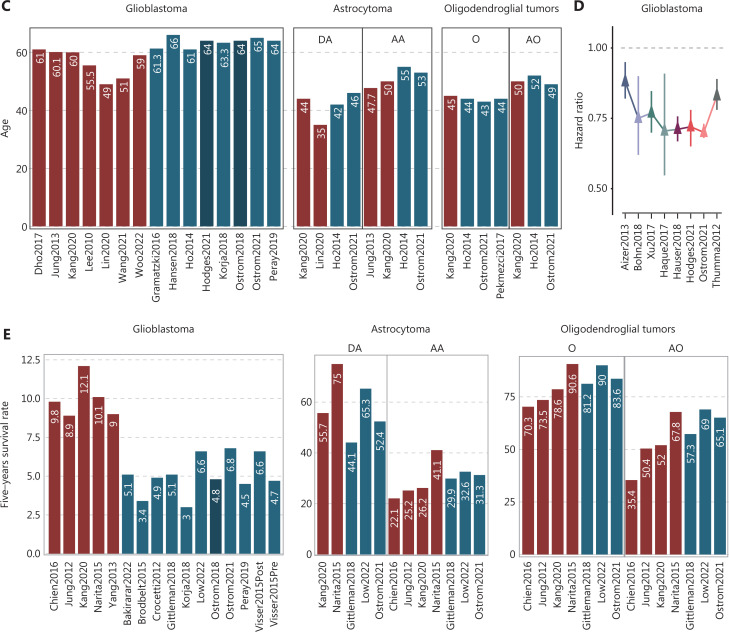
Clinical features of populations of East Asian and other ancestries. (A) Incidence rate of GBM, astrocytoma, and oligodendroglial tumors. Each bar represents the incidence rate from a study, with red for East Asians and blue for Whites. Within each subtype, all bars are sorted first by race, then by study. The exact incidence rate is also indicated. (B) Incidence rate ratio of gender (male:female) for subtypes from different studies. The percentages of males and females are plotted in pie charts, with corresponding male:female incidence ratios labeled for each study. (C) Median age at diagnosis of GBM, astrocytoma, and oligodendroglial tumors. Colors are as in (A). (D) Survival hazard ratio of API groups, with non-Hispanic White/White as the reference. (E) Five-year survival rates for GBM, astrocytoma, and oligodendroglial tumors for East Asian and mainly White populations. Colors are as in (A). DA, Diffuse Astrocytoma; AA, Anaplastic Astrocytoma; O, Oligodendroglioma; AO, Anaplastic Oligodendroglioma; EAS, East Asian; Visser2015Pre, Visser2015 before 2004; Visser2015Post, Visser2015 after 2004.

For GBM, the ASR was 4.71 for NHW and 2.00 for API in the USA (2000–2014), thus suggesting a limited effect of environmental factors and that the difference may be ancestry associated^16^. Independently, in a subsequent US epidemiological study (2014–2018), the ASR was 3.52^1^. The ASR was 4.64 in England (*n* = 10,743, 2007–2011)^32^ and 3.4 in Australia^33^. Similar ASR values have also been observed in other European countries, such as Switzerland (ASR = 3.9), France (ASR = 3.3), the Netherlands (ASR = 2.5), and Finland (ASR = 2.9)^34–37^, thus suggesting high ASR homogeneity among Whites. In contrast, a lower ASR among East Asians is supported by various studies. For example, the ASR was 0.92, 1.00, and 0.74 (crude incidence rate) in 3 Hong Kong, China Chinese population studies^38–40^. An ASR of 0.85 was reported in Taiwan, a province of China^19^. In Japan, the ASR was 1.26 in Miyazaki prefecture^30^. Four Korean studies reported ASR values of 0.59, 0.77, 0.87, and 1.11 (**Figure 2A**)^18,31,41,42^. Lower ASR in East Asians was still observed when only older adults (≥40 years of age) were considered^18^. In addition, all East Asian cohorts presented a lower ASR than the USA API group. Together, this evidence supports that NHW have an approximately fourfold higher GBM incidence rate than East Asians.

#### Astrocytoma

For astrocytoma, we used the grouping method introduced by Ostrom et al.^16^, mainly combining diffuse and anaplastic astrocytoma as non-GBM astrocytoma, and applied them to East Asian populations. According to the USA Surveillance, Epidemiology, and End Results Program (SEER) database, the ASR was 1.55 and 0.76 for NHW and API patients, respectively^16^. Furthermore, in a subsequent US cohort, the ASR was 1.4^1^. The ASR was 1.1 in the Netherlands^37^. Four Korean studies and one Chinese study supported a lower ASR among East Asian populations (ASR: 0.36–0.66) than NHW patients (ASR = 1.5) (**Figure 2A**)^18,19,31,41,42^. The ASR of Chinese (ASR = 0.63) and Korean (ASR = 0.66) patients was comparable to that of API (ASR = 0.76) patients^18,19^. The consistent patterns within regions that are predominantly either White or East Asian also suggest the limited influence of environmental factors on gliomagenesis.

#### Oligodendroglial tumors

The racial differences in ﻿oligodendroglial tumors, containing mainly oligodendroglioma and anaplastic oligodendroglioma, according to the grouping by Ostrom et al.^16^, follow a similar trend to those for GBM and astrocytoma, but are smaller in degree. In the USA, the ASR of NHW and API was 0.92 and 0.53, respectively^16^. An estimate from the EU27 (27 members of the European Union) indicated an ASR of 0.4^43^. Specifically in the Netherlands, the ASR was 0.6^37^. In contrast, the ASR ranged from 0.16 to 0.31 among East Asians (**Figure 2A**)^18,19,31,41,42^, values close to the API in the USA.

For oligodendroglial tumors and mixed gliomas, the ASR values in the USA (ASR = 0.56), Canada (ASR = 0.67), Western Europe (ASR = 0.61), Australia, and New Zealand (ASR = 0.64) were consistently higher than those in East Asia (ASR = 0.20) and Southeast Asia (ASR = 0.11), thus indicating high intra-racial homogeneity^44^. In summary, the ASR of oligodendroglial tumors for East Asians was lower than for Whites.

In conclusion, diffuse glioma in indigenous East Asians has a lower ASR than in Whites across all major subtypes. This difference persists between East Asians and Whites from the same geographical regions, thereby indicating the presence of genuine racial differences.

### Gender distribution and age of onset differences

Overall diffuse glioma shows a male preponderance^1,16,25^. The median age at diagnosis (MAD) is 63, 48, and 43 years for GBM, astrocytoma, and oligodendroglial tumors, respectively^16^. We performed a comparison of the gender distribution and MAD of East Asians compared with Whites for each subtype (**Figure 2B and 2C**).

#### GBM

Beyond the divergence in incidence, the GBM onset age also varies by race. The incidence has been found to be significantly (*P* < 0.005) higher (1.59-fold) in males than females in the USA among NHW^16^. Further studies in White populations also support a male prepondance^1,19,32,35–37,45^. In the Korean population, the IRRs (male-female ratio) were 1.31, 1.32, 1.27, 1.36^18,31,41,46^. Four studies based on Chinese have reported IRRs of 1.57, 1.69, 1.43, and 1.34 (**Figure 2B**)^19,38,39,47^. For each study, MADs were only reported for subtypes with sufficient patient numbers (*n* > 100). For GBM, the MAD of NHW (*n* = 128,976) and API (*n* = 2,929) was 64 and 61, respectively (2000–2014)^16^. Later, from 2014 to 2018, the MAD of the USA was 65^1,48^. Overall, a MAD of 64 or higher in Whites has been observed, except in studies in Zurich and the Netherlands^34–37,45^. In contrast, for East Asians, the MAD was 59 (*n* = 1010) in Hong Kong, China^39^. The MAD was 51 and approximately 49 in 2 hospital-based Chinese cohorts (**Figure 2C**)^49,50^. In 4 Korean studies, the MAD ranged from 55.5 to 61 (**Figure 2C**)^18,31,41,42,49^. In particular, the MAD was 60 in the largest Korean cohort (*n* = 5,796, 2007–2016), thus suggesting a MAD of 59–60 for East Asians^18^. In summary, the MAD is lower in East Asians than in NHW/Whites (3–9 year gap). An even more notable gap has been observed in hospital-based Chinese studies.

#### Astrocytomas

For astrocytomas, specifically diffuse and anaplastic astrocytomas, a male preponderance is also seen for the East Asian and the Whites (**Figure 2B**). The MAD of non-GBM astrocytomas was 50 and 41 for NHW and API^16^. A similar trend has also been observed for diffuse astrocytoma, with MAD values of 44, 35, 42, and 46 reported for South Korea, China (retrospective study), the Netherlands, and the USA, respectively^1,18,37,50^. Furthermore, in anaplastic astrocytoma, the MAD was 47.7 and 50 in 2 Korean populations, whereas the MAD was 53 and 55 for the USA and the Netherlands, respectively (**Figure 2C**)^1,18,42^. Overall, East Asians show a trend of lower MAD for anaplastic astrocytoma.

#### Oligodendroglial tumors

A male preponderance has been observed in the incidence of oligodendroglioma and anaplastic oligodendroglioma. Similarly, among NHW, a significantly higher male incidence has been observed for oligodendroglial tumors (**Figure 2B**). The MAD in oligodendroglial tumors, unlike other subtypes, showed minor differences between NHW (MAD = 44) and API (MAD = 41) in the USA^16^. Specifically, for oligodendroglioma, the MAD was 45 in the largest Korean cohort and was 43 and 44 among Whites^1,18,51^. For anaplastic oligodendroglioma, no consistent MAD difference has been observed between the East Asian and the Whites (**Figure 2C**)^1,18,37^. More compelling evidence is needed to validate a racial difference in MAD for oligodendroglial tumors.

In conclusion, GBM in East Asians has a lower MAD, and anaplastic astrocytoma in East Asians has shown a trend toward lower MAD. No clear racial MAD differences have been observed for oligodendroglial tumors. Adult diffuse gliomas are more common in males than females, and no significant gender distribution difference in GBM has been observed between East Asian and White populations.

### Survival outcomes

GBM is the most aggressive of diffuse gliomas, featuring the shortest 5-year relative survival rate (5-year RS), at 5.4% among US patients, followed by astrocytoma and oligodendroglial tumors, at 44.4% and 70.1%, respectively^16^. In this section, we compare the 5-year RS for each subtype between East Asian and White patients.

#### GBM

For GBM, the 5-year RS of USA API (8.8%) patients was longer than that of NHW patients (4.8%). For the USA population, the 5-year RS ranged from 6.6% to 6.8% (2014–2018)^1,25^. Moreover, API patients had a significantly (*P* < 0.01) lower hazard ratio (HR) (median HR = 0.74, range of HR: 0.70–0.88) than NHW patients (**Figure 2D**)^1,48,52–57^. The 5-year RS values for the 2 White populations in the US (2000–2014 and 2007–2018) were identical (5.1%)^58,59^. Independently, UK patients showed a 5-year RS of 3.4%, on the basis of 10,743 records (2007–2011)^32^. A lower value has also been observed in Finland (3%) and France (4.5%)^35,36^. In line with findings reported by Visser et al.^60^ [4.7% (1999–2001) and 6.6% (2005–2007) in 86 European registries], the 5-year RS was 4.9% among European Union members (EU27, 1995–2002)^43^. The homogeneity in the 5-year RS of Whites from the US and Europe revealed a consistent pattern of 5-year RS (approximately 5%). In contrast, a better 5-year RS has been observed in East Asians. For example, the 5-year RS was 9.8% (2002–2010) and 9% (2004–2010) in 2 Chinese populations^19,47^, and 10.1% in Japan (2001–2004)^20^. In Korea, the 5-year RS was 12.1% (*n* = 5,754, 2007–2016) and 8.9% (*n* = 1,676, 1999–2004) (**Figure 2E**)^18,46^. The difference between 12.1% and 8.9% might have arisen mainly from treatment improvements (such as the use of temozolomide treatment after 2004), thus indicating that even before the TMZ era, the Koreans had a 5-year RS of 8.9%^18,46^. All 5-year RS values of East Asians were close to or better than those of API. Thus on the basis of evidence from population-based and retrospective studies, East Asians show better survival than Whites for GBM.

#### Astrocytomas

For non-GBM astrocytomas, the 5-year RS between NHW (43.2%) and API (44.1%) groups in the USA were similar^16^. Specifically for diffuse astrocytoma, no consistent difference has been observed^1,18,20,25,59^. In addition, for anaplastic astrocytoma, the 5-year relative survival rate ranged from 25.2% to 26.2% in South Korea and was 22.1% in Taiwan, China, 41.1% in Japan, and 29.9% in the Whites^18–20,46,59^. The inconsistent patterns within East Asians suggest no survival differences in astrocytoma between NHW and East Asians (**Figure 2E**).

#### Oligodendroglial tumors

For oligodendroglial tumors, the 5-year RS between NHW (70%) and API (67.5%) groups in the USA was also similar^16^. For oligodendroglioma, the 5-year RS was 78.6% (*n* = 749) in Korea^18^. Better survival in Japan (90.6%), and poorer survival in Korea (73.5%) and Taiwan, China (70.3%) have been reported^19,20,46^. Likewise, the 5-year RS of Whites (81.2%–90%) was close to that of East Asians (**Figure 2E**)^1,25,59^. No consistent difference was observed for oligodendroglial tumors (**Figure 2E**)^18–20,46,59^.

In conclusion, better GBM survival in East Asian groups has been observed, but uncertainty remains for the other subtypes.

## Common germline SNPs derived from glioma GWASs

### GWASs for glioma

**Figure 3:**
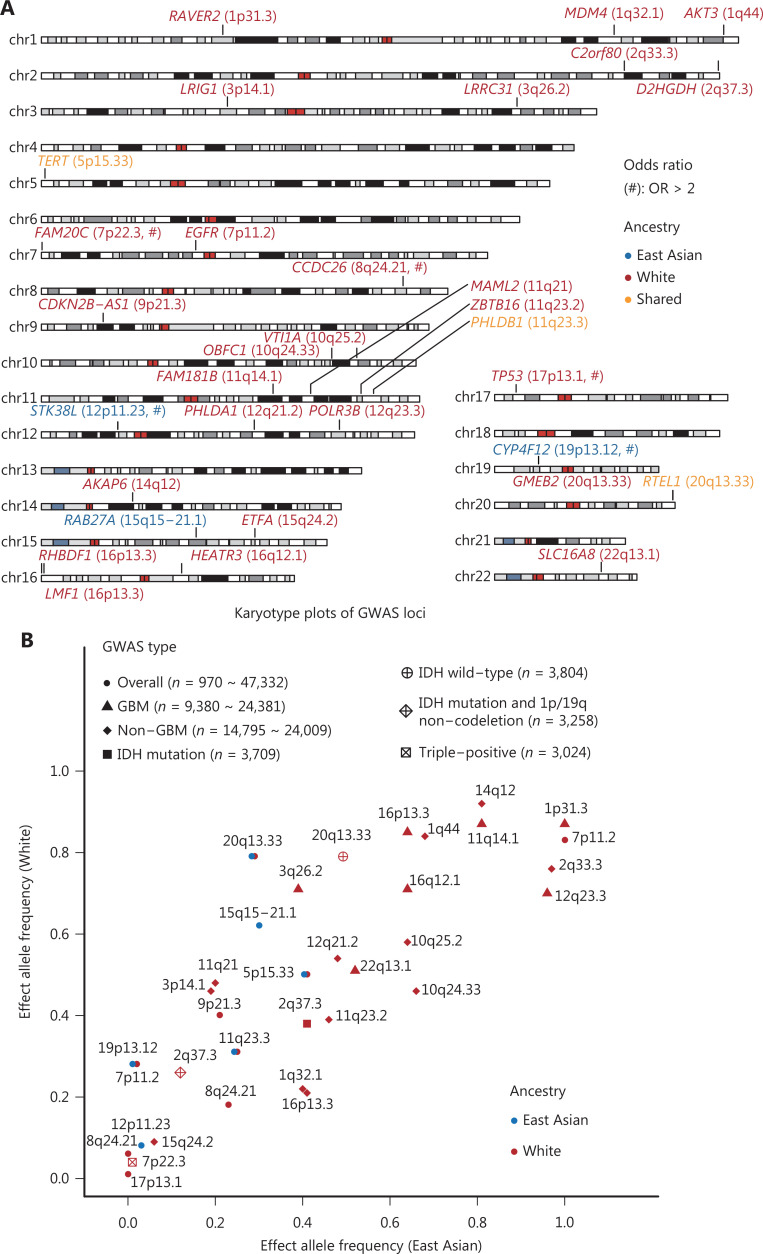
Summary of GWAS-reported genetic loci associated with glioma risk. (A) Karyotype plot of reported GWAS SNPs for adult diffuse glioma. All GWAS-identified risk SNPs from both the East Asian and White groups are plotted and labeled with gene name and locus in corresponding genome positions. Blue and red indicate SNPs unique to East Asian and White populations, respectively. Orange indicates SNPs identified or validated in both races. SNPs with an odds ratio (OR) >2 are labeled with the symbol #. (B) GWAS type and corresponding SNPs, tagged with cytoband. All SNPs from different GWAS were plotted according to effect allele frequencies from East Asian and White populations; the shape represents GWAS type, and the color represents ancestry. The ranges of sample numbers of each type of GWAS study are shown.

For GWASs, patients with East Asian ancestry have usually been from only East Asian countries or regions. To date, a total of 12 SNPs in 10 loci have been associated with the risk of pan-glioma, with odds ratios (ORs) ranging from 1.18 for rs2252586 (7p11.2; *EGFR*)^61^ and rs498872 (11q23.3; *PHLDB1*)^62^ to 3.55 for rs688755 (19p13.12; *CYP4F12*)^63^. Among these variants, 9 SNPs in 7 loci (5p15.33, 7p11.2, 8q24.21, 9p21.3, 11q23.3, 17p13.1, and 20q13.33) have been identified from White populations, and most show significant racial differences in effect allele frequency (EAF) between East Asian and White populations (*P* for chi-square test < 0.05; **Table 1** and **Figure 3**). Notably, both rs78378222 (17p13.1; *TP53*)^64^ and rs55705857 (8q24.21; *CCDC26*)^65^ are considered potential White-specific SNPs, with an EAF of 0 among East Asian populations.

**Table 1:** Summary of GWAS-reported genetic loci associated with glioma risk

Author (year)	Ancestry	Type	SNP	Locus	Nearby gene	Annotation^a^	Allele^b^	EAF^c^	*P* ^d^	OR_GWAS_^e^	*P*_GWAS_^e^	PMID^f^
Melin (2017)	White	GBM	rs12752552	1p31.3	*RAVER2*	Intronic	T/C	1.00/0.87	2.18E-32	1.22	2.04E-09	28346443
Melin (2017)	White	Non-GBM	rs4252707	1q32.1	*MDM4*	Intronic	A/G	0.40/0.22	1.06E-18	1.19	3.34E-09	28346443
Melin (2017)	White	Non-GBM	rs12076373	1q44	*AKT3*	Intronic	G/C	0.68/0.84	7.38E-16	1.23	2.63E-10	28346443
Melin (2017)	White	Non-GBM	rs7572263	2q33.3	*C2orf80*	Intronic	A/G	0.97/0.76	2.05E-45	1.20	2.18E-10	28346443
Eckel-Passow (2020)	White	*IDH* mutation	rs5839764	2q37.3	*D2HGDH*	Intronic	G/C	0.41/0.38	0.215	1.51/1.56	2.82E-10	32386320
Eckel-Passow (2020)	White	*IDH* mutation and 1p/19q non-codeletion	rs1106639	2q37.3	*D2HGDH*	Missense	A/G	0.12/0.26	2.82E-15	1.71/1.72	4.96E-08	32386320
Melin (2017)	White	Non-GBM	rs11706832	3p14.1	*LRIG1*	Intronic	C/A	0.19/0.46	6.02E-37	1.15	7.66E-09	28346443
Walsh (2014)	White	GBM	rs1920116	3q26.2	*LRRC31*	Intronic	G/A	0.39/0.71	1.90E-47	1.30	8.30E-09	24908248
Shete (2009)	White	Overall	rs2736100	5p15.33	*TERT*	Intronic	G/T	0.41/0.50	1.46E-04	1.27	1.50E-17	19578367
Chen (2019)	East Asian	Overall	rs2736100	5p15.33	*TERT*	Intronic	G/T	0.41/0.50	1.46E-04	1.27	2.45E-12	30714141
Eckel-Passow (2020)	White	Triple-positive	rs111976262	7p22.3	*FAM20C*	Intergenic	A/C	0.01/0.04	6.55E-05	3.52/3.05	9.56E-09	32386320
Sanson (2011)	White	Overall	rs11979158	7p11.2	*EGFR*	Intronic	A/G	1.00/0.83	5.41E-41	1.23	7.72E-08	21531791
Sanson (2011)	White	Overall	rs2252586	7p11.2	*EGFR*	Intergenic	T/C	0.02/0.28	3.94E-61	1.18	2.09E-08	21531791
Jenkins (2012)	White	Overall	rs55705857	8q24.21	*CCDC26*	Intergenic	G/A	0.00/0.06	1.77E-14	3.11	5.00E-25	22922872
Shete (2009)	White	Overall	rs4295627	8q24.21	*CCDC26*	Intergenic	G/T	0.23/0.18	4.99E-03	1.36	2.34E-18	19578367
Shete (2009)	White	Overall	rs4977756	9p21.3	*CDKN2B-AS1*	Intronic	G/A	0.21/0.40	2.31E-21	1.24	7.24E-15	19578367
Melin (2017)	White	Non-GBM	rs11598018	10q24.33	*OBFC1*	Intronic	C/A	0.66/0.46	6.45E-19	1.14	3.39E-08	28346443
Kinnersley (2015)	White	Non-GBM	rs11196067	10q25.2	*VTI1A*	Intronic	A/T	0.64/0.58	7.30E-03	1.19	4.32E-08	26424050
Melin (2017)	White	GBM	rs11233250	11q14.1	*FAM181B*	Intergenic	C/T	0.81/0.87	8.72E-04	1.24	9.95E-10	28346443
Melin (2017)	White	Non-GBM	rs7107785	11q21	*MAML2*	Intronic	T/C	0.20/0.48	1.01E-40	1.16	3.87E-10	28346443
Kinnersley (2015)	White	Non-GBM	rs648044	11q23.2	*ZBTB16*	Intronic	T/C	0.46/0.39	2.46E-03	1.25	6.26E-11	26424050
Shete (2009)	White	Overall	rs498872	11q23.3	*PHLDB1*	5'-UTR	T/C	0.25/0.31	5.80E-03	1.18	1.07E-08	19578367
Chen (2019)	East Asian	Overall	rs498872	11q23.3	*PHLDB1*	5'-UTR	A/G	0.25/0.31	5.80E-03	1.25	3.41E-09	30714141
Chen (2019)	East Asian	Overall	rs10842893	12p11.23	*STK38L*	Intronic	T/C	0.03/0.08	2.99E-05	2.07	2.33E-12	30714141
Kinnersley (2015)	White	Non-GBM	rs12230172	12q21.2	*PHLDA1*	Intergenic	G/A	0.48/0.54	6.52E-03	1.23	7.53E-11	26424050
Kinnersley (2015)	White	GBM	rs3851634	12q23.3	*POLR3B*	Intronic	T/C	0.96/0.70	1.59E-51	1.23	3.02E-09	26424050
Melin (2017)	White	Non-GBM	rs10131032	14q12	*AKAP6*	Intronic	G/A	0.81/0.92	1.56E-11	1.33	5.07E-11	28346443
Chen (2019)	East Asian	Overall	rs4774756	15q15-21.1	*RAB27A*	Intronic	C/A	0.30/0.62	9.52E-47	1.24	6.12E-08	30714141
Kinnersley (2015)	White	Non-GBM	rs1801591	15q24.2	*ETFA*	Missense	A/G	0.06/0.09	1.00E-02	1.36	5.71E-09	26424050
Melin (2017)	White	GBM	rs2562152	16p13.3	*RHBDF1*	Intergenic	T/A	0.64/0.85	7.30E-28	1.21	1.93E-08	28346443
Melin (2017)	White	Non-GBM	rs3751667	16p13.3	*LMF1*	Synonymous	T/C	0.41/0.21	2.78E-23	1.18	2.61E-09	28346443
Melin (2017)	White	GBM	rs10852606	16q12.1	*HEATR3*	Intronic	C/T	0.64/0.71	7.95E-04	1.18	1.29E-11	28346443
Stacey (2011)	White	Overall	rs78378222	17p13.1	*TP53*	3'-UTR	C/A	0.00/0.01	0.157	2.35	1.00E-05	21946351
Li (2021)	East Asian	Overall	rs688755	19p13.12	*CYP4F12*	Synonymous	C/T	0.01/0.28	8.42E-63	3.55	2.35E-08	34319593
Shete (2009)	White	Overall	rs6010620	20q13.33	*RTEL1*	Intronic	G/A	0.29/0.79	4.97E-113	1.28	2.52E-12	19578367
Chen (2019)	East Asian	Overall	rs6010620	20q13.33	*RTEL1*	Intronic	G/A	0.29/0.79	4.97E-113	1.29	7.39E-12	30714141
Eckel-Passow (2020)	White	*IDH* wild-type	rs4809313	20q13.33	*GMEB2*	Intronic	G/A	0.49/0.79	1.02E-45	1.51/1.59	2.60E-10	32386320
Melin (2017)	White	GBM	rs2235573	22q13.1	*SLC16A8*	Synonymous	G/A	0.52/0.51	6.57E-01	1.15	1.76E-10	28346443

^a^Annotated by HaploReg website (https://pubs.broadinstitute.org/mammals/haploreg/haploreg.php).

^b^Allele, effect allele/reference allele.

^c^EAF, effect allele frequency (East Asian/White population) in the 1000 Genomes project.

^d^Chi-square test for the difference in EAF between East Asian and White populations.

^e^Derived from previous glioma GWASs.

^f^PubMed ID.

Note: Overall, defined by glioma; GBM, defined by glioblastoma; triple-positive, defined by *IDH* mutation, *TERT* mutation and 1p19q co-deletion.

Chen et al.^66^ performed the first multi-stage glioma GWAS among a Han Chinese population in 2019, on the basis of data from 992 cases and 1,008 controls from Shanghai and Beijing, and a subsequent replication stage using 2,105 cases and 3,374 controls from Shanghai, Nanjing, Beijing, and Xiàn. Interestingly, the authors have validated 3 White-reported glioma risk loci in the Chinese population: rs2736100 (OR_random-effect_ = 1.27; 5p15.33; *TERT*), rs498872 (OR_fixed-effect_ = 1.25; 11q23.3; *PHLDB1*), and rs6010620 (OR_fixed-effect_ = 1.29; 20q13.33; *RTEL1*). Therefore, these 3 SNPs are potential trans-ancestry risk loci for glioma. In addition, Chen et al.^66^ have identified one novel glioma risk-associated locus on 12p11.23 (rs10842893; OR_fixed-effect_ = 2.07; *STK38L*) as well as a suggestive association at 15q15-21.1 (rs4774756; OR_fixed-effect_ = 1.24; *RAB27A*) among the Chinese population. In a recent study, Li and colleagues have performed a Chinese GWAS for glioma with 485 cases and 485 healthy controls and found a significant East Asian specific low-frequency variant (EAF_East Asian_ = 0.01; EAF_White_ = 0.28) with a large effect on 19p13.12 (rs688755; OR = 3.55; *CYP4F12*)^63^.

For the 3 East Asian GWAS-identified risk loci (i.e., 12p11.23, 15q15-21.1, and 19p13.12), the risk SNP (i.e., rs10842893) located on 12p11.23 was in the intronic region of the gene *STK38L*, and the expression of *STK38L* was higher in the glioma samples than the normal samples in TCGA database. The rs4774756 SNP at 15q15-21.1 is located within the intronic region of *RAB27A*, a gene encoding a member of the Rab small GTPase family. Several studies have shown that Rab27a promotes proliferation and invasion, and represses apoptosis, on the basis of functional assays in glioma cell lines^67,68^. Another risk SNP (i.e., rs688755) located on 19p13.12 is near the genes *CYP4F12*, encoding a protein that oxidizes arachidonic acid; *PGE2*, encoding the omega-side chain of prostaglandin E2; and *PGH2*, encoding prostaglandin H2^69^. Several studies have shown that *PGE2* increases the survival, migration, and proliferation of glioma cells, thus indicating the critical role of *CYP4F12* and *PGE2* in the development of glioma^70^. Nevertheless, further functional evaluations are needed to elucidate the roles of these SNPs and nearby genes to understand the development of glioma.

### GWASs for histological subtypes of glioma

GWASs stratified by histological entity have identified novel germline variants associated with GBM and non-GBM, in addition to overall glioma,^71–73^. These newly identified GBM and non-GBM-specific variants have not reached genome-wide significance for overall glioma risk, thus revealing potential heterogeneity among glioma histological subtypes.

For GBM, a total of 7 SNPs have been reported to be associated with the risk of GBM (effect sizes ranging from 1.15 to 1.3) in White populations, including rs12752552 (1p31.3; *RAVER2*), rs1920116 (3q26.2; *LRRC31*), rs11233250 (11q14.1; *FAM181B*), rs3851634 (12q23.3; *POLR3B*), rs2562152 (16p13.3; *RHBDF1*), rs10852606 (16q12.1; *HEATR3*), and rs2235573 (22q13.1; *SLC16A8*). In agreement with the findings from overall glioma-reported loci, most of these SNPs show clear differences in allele frequency among ancestries. Li et al.^63^ have also evaluated the effects of these SNPs in a Chinese population but observed no significant association. In contrast, a non-GBM GWAS has identified 12 genetic loci (ORs ranging from 1.14 to 1.36) among White populations, including rs4252707 (1q32.1; *MDM4*), rs12076373 (1q44; *AKT3*), rs7572263 (2q33.3; near *IDH1*), rs11706832 (3p14.1; *LRIG1*), rs11598018 (10q24.33; *OBFC1*), rs11196067 (10q25.2; *VTI1A*), rs7107785 (11q21; *MAML2*), rs648044 (11q23.2; *ZBTB16*), rs12230172 (12q21.2; *PHLDA1*), rs10131032 (14q12; *AKAP6*), rs1801591 (15q24.2; *ETFA*), and rs3751667 (16p13.3; *LMF1*). Similarly to the results in GBM, findings by Li et al.^63^ have not replicated the risk effects of these SNPs in the Chinese population.

Comparison of the genetic loci explicitly associated with the risk of GBM and non-GBM has indicated only one shared region (16p13.3), thereby demonstrating the differences in the functions of genetic variants involved in the development of glioma with different histological features. Further large glioma GWASs with multiple ancestries and refined subtype classifications remain needed.

### GWASs for molecular subtypes of glioma

Although glioma cases have been divided into GBM and non-GBM classifications for discovering novel histological subtype-specific risk loci, glioma is increasingly being understood to have several distinctive molecular subtypes, such as *IDH* mutation, arm level 1p/19q co-deletion, and promoter mutation in the telomerase reverse transcriptase (*TERT*) gene^4^. Eckel-Passow et al.^74^ have evaluated the associations of previously reported risk SNPs with the risk of molecular subtype-specific glioma; interestingly, most have shown significant associations with *IDH*-mutant glioma risk.

Eckel-Passow et al.^75^ have also performed a large-scale glioma GWAS stratified by molecular subtypes defined by combinations of *IDH* mutation, 1p/19q co-deletion, and promoter mutation in *TERT* among White populations. Notably, they have identified 2 novel genetic loci and a GWAS-reported region associated with the risk of specific glioma molecular subtypes: rs5839764 (2q37.3; *D2HGDH*) for *IDH* mutation; rs1106639 (2q37.3; *D2HGDH*) for *IDH* mutation and 1p/19q non-codeletion; rs111976262 (7p22.3; *FAM20C*) for triple-positive (*IDH* mutation, *TERT* mutation and 1p19q co-deletion); and rs4809313 (20q13.33; *GMEB2*) for *IDH* wild-type. However, whether these molecular subtype-specific variants will be validated in East Asian populations remains unclear.

### Common germline SNPs derived from candidate gene or pathway approaches

Beyond GWAS analysis, the candidate gene or pathway approach is another effective method to identify genetic loci associated with glioma risk. As shown in **Supplementary Table S2**, we collected glioma risk-associated SNPs identified in candidate gene studies in the past decade, thus providing additional novel SNPs (e.g., 2q23.3 and 3q22.2) beyond those from GWASs. Most SNPs were found in Chinese populations. However, most candidate SNPs were identified in limited sample sizes (usually fewer than 2,000), with borderline significance and without external validation. Therefore, the associations of these SNPs with the risk of glioma must be further validated.

### Application of GWAS variants in glioma risk prediction

Although GWASs have identified multiple risk variants associated with glioma development, applying these variants in clinical practice remains challenging. Here, the essential clinical value of GWAS-reported SNPs is prediction of the risk of developing glioma^76^, and identification of individuals at high risk to support early prevention.

Recently, Choi et al.^77^ have evaluated the clinical utility of 25 GWAS-identified glioma risk loci in in a large-scale UK Biobank cohort comprising 400,807 participants of White ancestry. During a follow-up spanning a median of 5.8 years, 312 incident glioma cases were distinguished in this cohort. Interestingly, after construction of a polygenic risk score based on these GWAS SNPs, the authors found that the risk score had sufficient discriminatory ability to distinguish people with and without glioma, with an area under the receiver operating characteristic curve of 0.64. Compared with individuals in the middle polygenic risk score quintile (40%–60%), those in the top 5% had a 2.55-fold greater risk of glioma. In comparison, those in the bottom 5% had an approximately 69% lower risk of glioma. These results suggested that GWAS-reported germline variants can be applied in identifying high-risk individuals for early glioma prevention.

In addition to predicting the risk of overall glioma, Eckel-Passow et al.^74^ have found that the GWAS SNPs-based risk score can be used to predict the risk of molecular subtype-specific glioma, on the basis of discovery (1,273 glioma cases and 443 controls) and validation (852 glioma cases and 231 controls) datasets from a White population. For example, compared with participants in the median quantile (45%–55%) of the risk score, those in the highest 5% had a more than 14-, 19- and a 5-fold increase in the relative risk of developing *IDH* mutant and 1p/19q codeleted, *IDH* mutant and 1p/19q non-codeleted, and *IDH* wild-type glioma, respectively. Notably, the authors have also found that those germline variants can be used to predict glioma molecular subtype, such as *IDH* mutation status, with a concordance index of 0.85. These results further demonstrate the potential value of germline SNPs in guiding clinical decision-making.

However, given that most GWAS-identified SNPs were derived from White populations, whether these germline SNPs have sufficient ability to predict the risk of overall or subtype-specific glioma in East Asian populations remains unknown. Eckel-Passow et al.^76^ have evaluated the predictive power of the White-derived glioma polygenic risk model in independent cohorts of White and non-White ancestries (e.g., East Asian and African populations). They have found that White-based risk scores do not generalize across ancestries, thus demonstrating that genetic studies must be performed on more diverse populations, particularly for East Asians.

## Somatic mutation profile differences

Studies are increasingly investigating the genomic landscape of glioma by including more East Asian populations to fill gaps in the genetic diversity of data^49,78–80^. Notably, the Chinese Glioma Genome Atlas has collected, archived, and shared large-scale glioma omics data from the Chinese population, thus aiding in the ease and integration of studies of East Asian glioma^81^. Currently, the racial differences regarding somatic profiles have been illustrated for some cancers. For example, for lung adenocarcinoma, Zhang et al.^82^ have identified that the percentage of Native American ancestry is positively correlated with *EGFR* somatic mutations. Attempts to identify genetic pathways showing racial disparities have been put into practice for glioma^83,84^. Nonetheless, studies have been limited by sample sizes, and the differences for glioma have not been well summarized in previous studies. Here, we reviewed the racial differences in somatic profiles (**Table 2**). Because East Asians were under-represented in the previous TCGA program comprising mainly White ancestry populations, the comparison of somatic profiles was based mainly on East Asians from East Asian geographical regions and people of White ancestry.

**Table 2:** Summary of somatic differences between East Asian (EAS) and White ancestries

Study	Data source^a^	Subtype	#Total	#EAS	Comparison^b^	EAS enriched	White enriched	Shared pattern
Koo (2020)	South Korea	GBM	340	90	TCGA	MAPK and p53 pathways	/	/
Chan (2016)	China	LGG	459	459	TCGA	Triple-negative gliomas	/	/
Zeng (2021)	China	HGG+LGG	83	83	MSKCC	*H3F3A* mutation, *MET* amplification	*TERT* mutation, *EGFR*, and *CDKN2A/B* CNV	/
Hu (2018)	China & South Korea	sGBM	188	108	TCGA	/	Hypermutation	/
Lassman (2019)	China, Singapore, South Korea	GBM	3150	484	Enrolled White	/	*EGFR* amplification	/
Suzuki (2015)	Japan	LGG	760	335	TCGA	*SETD2* somatic mutation	/	Three-fourths *IDH1* mutation

^a^Country where the samples were collected.

^b^Population compared with East Asians.

Note: LGG, lower grade glioma; HGG, high-grade glioma; sGBM, secondary GBM

Leveraging the panel sequencing data of 83 Chinese glioma samples, Zeng et al.^24^ have identified significant differences in somatic mutations between the Chinese database and the Memorial Sloan Kettering Cancer Center (MSKCC) database comprising mainly individuals of White ancestry. The *H3F3A* somatic mutation and *MET* amplification are enriched in the Chinese cohort. In contrast, the *TERT* and *EGFR* somatic mutations and *CDKN2A/B* copy number alterations are significantly depleted in the Chinese glioma cohort (*P* < 0.05) (**Table 2**). Because the Chinese cohort included 4 grades of glioma, and *H3F3A* was a marker of pediatric glioma, the enrichment or depletion of gene somatic mutations might have been biased by the grade or subtype^24^.

Focusing on non-GBM, by including 332 lower-grade gliomas (LGGs) from a Japanese cohort, compared with a total of 425 samples in TCGA, Suzuki et al.^22^ have identified a similar frequency of *IDH1/2* mutation in the 2 cohorts, reaching 78.01% in the Japanese cohort and 80.47% in TCGA. The *TP53* mutation frequency was slightly lower in the Japanese cohort (40.36%) than TCGA (49.65%), whereas both the *SETD2* mutation rate and *TERT* promoter mutation detection rate were lower in TCGA. The *TERT* mutation depletion might have been due to the low sequencing coverage^22^. In addition, the Japanese population and TCGA cohort showed no significant age distribution differences stratified by disease stage. Meanwhile, as described by Chan et al.^23^, for LGG, triple-negative (1p/19q non-codeletion, *IDH*, and *TERT* wild-type) glioma showed a higher frequency in the Chinese population (17.4%) than that in the TCGA population (7.0%) (**Table 1**)^85^. Among the triple-negative gliomas within the Chinese population, both *TP53* and *H3F3A* wild-type patients have been predicted to have significantly better survival than the *TP53* and *H3F3A* mutant patients; therefore, further examination of the somatic status might help achieve better prognosis or therapy strategies.

GBM has also been evaluated in a large cohort of 3303 patients initially recruited for *EGFR* amplification screening for an *EGFR* antibody clinical trial. *EGFR* amplification, as detected by fluorescence *in situ* hybridization, had a higher frequency in the overall population (approximately 56%) and a significantly lower frequency in East Asians (approximately 35%); this finding was independently validated in a self-reported cohort of 153 Japanese individuals (33% *EGFR* amplification) (**Table 2**)^86^. The classical glioma subtype in the Chinese population does not show consistent strong *EGFR* expression^87^. By focusing on GBM whole exome sequencing data in 90 Korean patients, Harim et al.^21^ have identified greater enrichment of the P53 pathway with respect to that in TCGA cohort (*n* = 250), although *EGFR* amplification was not described (**Table 2**). Overall, the weakness of *EGFR*-associated features may be unique to East Asian populations. Moreover, a study of 188 patients with secondary GBM has indicated enriched hypermutation in the White group, an effect possibly associated with broad TMZ treatment in that group^78^.

## Discussion and future directions

Although the differences in glioma incidence, mortality, and survival outcomes between East Asian and White/NHW patients might have been confounded by different environmental factors, the difference persists and remains valid, according to results from East Asian populations living in the USA and the UK, thus partially controlling for environmental factors^44^.

Although glioma-associated factors have been well summarized in Whites^88^, factors specific to East Asians have not been well addressed. It was reported that smoking and height might be associated with glioma onset in Korean studies^89–91^, and the atomic bomb was reported to be associated with glioma risk by ﻿Radiation Effects Research Foundation in Japan^92^. However, whether these environmental or behavioral factors are associated with glioma exclusively in East Asians remained to be determined.

Mortality rate is a critical indicator of the effects of treatment intervention. The glioma incidence-based mortality rates during 1995–2018 were calculated for White populations independently each year, and the median value was 5.155. However, the diffuse glioma mortality rate among East Asians has rarely been precisely determined, and most studies have evaluated mortality for malignant brain cancer and other CNS tumors as a whole^93^ and reported a mortality of 4.42 and 4.43 per 100,000 per year^1,26^. Because GBM is the most malignant subtype, with a 5%–12% 5-year RS, and accounts for 58.4% of gliomas^1^, the incidence data might be a crude estimate of the mortality data^27^. Nonetheless, for other low-grade subtypes, detailed statistics of mortality by race are needed for disease surveillance and policymaking. Future inclusion of the Chinese database might provide a more comprehensive portrayal of adult diffuse glioma mortality among East Asians.

In reviewing the overall incidence rate for “adult diffuse glioma”, for which data stratified by race are unavailable, a comprehensive estimation has been conducted for “glioma” instead. Considering that TMZ treatment is a known factor of better survival outcomes, it should be considered as a confounding factor when comparing survival differences between East Asians and Whites^94^. In another study based on 205 Chinese individuals (median survival time: 12.0 months) from 1999–2004, no additional survival benefit was observed beyond that in Western patients (median survival time: 12.1 months) in a clinical trial. This finding might have been due to a lack of access to temozolomide or other chemotherapy treatment. Furthermore, the lower age distribution (MAD = 57) of the Western patients enrolled in the clinical trial might have prolonged the survival^94,95^. In Hong Kong, China the 5-year RS of 3% (*n* = 1,010, 2006–2019) requires further verification^39^. Future hospital-based registries might help eliminate potential confounding factors such as TMZ treatment in addressing racial survival differences. Because most enrolled studies were from the TMZ era (after 2004), TMZ treatment might not be sufficient to explain the survival differences between races^28^. Even among studies based on records before 2004, the 5-year RS of East Asians [8.9% in Korea (1999–2004)] exceeded that among Whites [4.9% in White (1995–2002); 3.9% in NHW from the USA (2000–2004)]^16,43,46^.

Although we compared East Asians mainly with NHW or Whites, compromises were necessary when data for NHW or Whites were unavailable. For example, the MAD from the entire SEER database (comprising approximately 90% White individuals) have been presented as an estimation of astrocytoma and ﻿oligodendroglial tumors among Whites; such estimation should be feasible when the database records are large^1^. However, no explicit conclusion can be drawn. Future studies excluding non-adult samples remain needed to make further claims. Moreover, the 5-year RS among Whites was included for all subtypes and compared with that among East Asians (**Figure 2D**). Racial differences have been observed for different groups within the USA and UK. Populations of East Asian descent and those of White ancestry show homogeneity in different countries. These findings should be informative in addressing racial differences. To provide quantitative comparisons, examination of only East Asians in the SEER database might help verify the differences by considering confounding factors. Nonetheless, in GBM, consistent evidence supports a lower incidence rate, early onset age, and prolonged survival for East Asians than that in the White ancestry.

Multiple glioma risk loci have been identified; however, the potential molecular mechanisms underlying these associations remain unclear, indicating the critical role of functional genomics in the post-GWAS era ^96,97^. Many of these risk SNPs are located in non-coding regions, thus suggesting that genetic effects may arise from regulation of the expression of nearby genes^98^. Therefore, exploration of which genes are affected by the germline variants and how changes in the function or regulation of the target genes lead to the development of glioma is urgently needed.

The genetic architecture of glioma between White and East Asian populations may differ. Most of the glioma GWASs published to date have been performed to identify glioma risk loci in White populations. However, GWASs in non-White (particularly East Asian) populations remain lacking. As evidenced in this review, only 6 risk loci (i.e., 5p15.33, 11q23.3, 20q13.33, 12p11.23, 15q15-21.1, and 19p13.12) have been reported to be associated with glioma risk at a genome-wide significant level in East Asian (i.e., Chinese) population, of which 3 (5p15.33, 11q23.3, and 20q13.33) were also derived from White populations. Further overall and subtype-specific glioma GWASs in non-White populations, including East Asian populations, are thus needed.

Cumulative evidence has confirmed the utility of germline variants in clinical practice; these variants may serve as a more robust and cost-efficient tool for disease risk stratification than other risk factors or biomarkers^99,100^. For example, SNPs do not change over time and thus require measurement only once. Previous studies in White populations have demonstrated that GWAS-identified SNPs have sufficient predictive power to identify individuals at high risk of developing glioma and their molecular subtypes^74^. However, whether these germline variants derived from White populations can be used in East Asian populations remains to be determined^101^. Importantly, White-specific germline SNPs have shown less discriminatory ability in non-White populations^102^; therefore, further studies on the development and independent validation of a genetic risk prediction model for glioma in East Asian populations are needed.

Multiple studies have identified the potential inherited genetic architecture in somatic alterations^103^. For example, Carter et al.^104^ have applied a pan-cancer analysis to validate more than 400 genetic interactions between germline SNPs and somatic events (e.g., somatic alteration of specific cancer genes). Sun et al.^105^ have identified multiple germline genetic variants associated with tumor mutational burden, particularly cancer types. Therefore, whether some somatic alterations in glioma, such as *IDH* mutation and 1p/19q co-deletion, are correlated with germline variants must be explored in the future, to help researchers better understand the molecular mechanisms of tumorigenesis.

Identifying potential divergent somatic profiles between East Asians and White populations might aid in identifying new markers and delineating glioma tumorigenesis and development, and enabling personalized treatments. Meanwhile, several studies based on East Asian populations^106–111^ and other studies involving White-only populations^112–120^ are available for further evaluation. The majority of these studies were performed independently either in East Asian or White populations. Thus a comprehensive integration of DNA/RNA sequencing data from these studies is needed to investigate the differences between East Asians and other racial groups after controlling for confounding factors, such as disease stage, subtype, sequencing protocols, or batch effects. Future repurposing of these independent large-scale data might ultimately pave the way to innovative discoveries that may help explain the divergence in onset age, and survival differences among ancestries, and elucidate disease mechanisms.

Several study limitations must be noted. First, we could not ensure that all patients registered in East Asian countries or regions were of Asian ancestry, although the likelihood of inclusion of non-East Asians is very low in these countries. In addition, because East Asian, Southeast Asian, and Indian populations showed similar diffuse glioma incidence rates lower than those in Whites, we believe that, despite the unknown proportion of East Asians in the API group, high homogeneity within these different Asian groups exists, thus suggesting that API provide an acceptable representation of East Asians^44^. Second, although Taiwan, China, and Hong Kong, China should represent the Chinese population well, the mainland China cohort was under-represented because of the lack of a comprehensive prospective national-wide glioma database. Only retrospective studies were retrieved for this review, but the National Brain Tumor Registry of China is collecting data from 2019 to 2024; a future large-scale database could aid in the delineation of glioma in the Chinese population^121^. The upcoming China brain registry, with a detailed record of CNS tumors in the Global Burden of Disease and the Global Cancer Observatory, might also help address this problem. Third, the NHW/White population epidemiological data might not have completely corresponded to the White ancestry data reviewed in the GWAS and somatic analyses. In addition, because various studies used different subgrouping criteria for diffuse glioma, drawing direct comparisons across populations was difficult. Finally, the most recent 5^th^ edition of the WHO classification of CNS tumors highlights the deficiencies in relying on incomplete historical glioma molecular data to draw definitive conclusions^4^. Population-based brain tumor registries should contain as much molecular information as possible to accommodate subsequent new diagnostic criteria to enable more reliable comparisons.

## Conclusions

This study comprehensively reviewed the epidemiology and genomic data of adult diffuse gliomas among East Asian populations. We summarized the features of diffuse glioma among East Asians and compared them with those in existing population studies predominantly in people of White ancestry. We demonstrated a lower incidence rate of adult diffuse glioma, earlier onset age for GBM, and prolonged overall survival in East Asian populations with GBM than NHW/White populations. Apart from several GWAS SNPs found only in East Asians, we identified several somatic mutations enriched or depleted in the East Asian group. Despite several independent studies on East Asians, the differences between East Asians and other races remain poorly surveyed. Uncovering and characterizing the characteristics of diverse populations may help pave the way to further investigation of how ancestral background contributes to gliomagenesis and clinical outcomes.
