# Supplementary material for: Epidemiological characteristics and genetic alterations in adult diffuse glioma in East Asian populations
Source: Cancer Biol Med. 2022 Nov 1;19(10):1440–59. doi: 10.20892/j.issn.2095-3941.2022.0418 (PMC9630523; doi:10.20892/j.issn.2095-3941.2022.0418)
Supplement: Supplementary file 1 [file cbm-19-1440-s001.zip › cbm-19-1440-s002.docx]

**Supplementary Table S2.** Summary of previously reported genetic loci associated with glioma risk derived from candidate gene or pathway studies.

| Author (year) | Population | N | SNP | Type | Nearby gene | Loci | Allele^a^ | Model | OR | *P* | PMID^b^ |
| --- | --- | --- | --- | --- | --- | --- | --- | --- | --- | --- | --- |
| Meng (2015) | Chinese | 1915 | rs2278089 | Overall | NMI | 2q23.3 | C/A | Dominant | 1.57 (1.29-1.90) | 4.23E-06 | 25387807 |
| Meng (2015) | Chinese | 1915 | rs2194492 | Overall | NMI | 2q23.3 | C/G | Dominant | 1.49 (1.22-1.82) | 1.20E-04 | 25387807 |
| Meng (2015) | Chinese | 1915 | rs6734376 | Overall | NMI | 2q23.3 | G/A | Dominant | 0.06 (0.03-0.13) | 8.65E-13 | 25387807 |
| Meng (2015) | Chinese | 1915 | rs3854012 | Overall | NMI | 2q23.3 | A/G | Recessive | 0.54 (0.41-0.70) | 4.64E-06 | 25387807 |
| Meng (2015) | Chinese | 1915 | rs11730 | Overall | NMI | 2q23.3 | C/T | Recessive | 0.60 (0.47-0.78) | 1.50E-04 | 25387807 |
| Qin (2015) | Chinese | 523 | rs3791679 | Overall | EFEMP1 | 2p16.1 | A/G | Dominant | 1.55 (1.04-2.32) | 0.02 | 26823870 |
| Wang (2021) | Chinese | 744 | rs2239611 | Astrocytoma | ST6GAL1 | 3q27.3 | A/G | Dominant | 0.74 (0.55-1.00) | 0.049 | 33836687 |
| Mahjabeen (2021) | Pakistan | 470 | rs9809619 | Overall | CEP-63 | 3q22.2 | T/G | Additive | 5.11 (3.65-7.14) | < 0.001 | 34156311 |
| Mahjabeen (2021) | Pakistan | 470 | rs13060247 | Overall | CEP-63 | 3q22.2 | T/C | Additive | 3.73 (2.83-4.91) | < 0.001 | 34156311 |
| Chen (2019) | Chinese | 744 | rs2378456 | Astrocytoma | LPP | 3q28 | C/G | Recessive | 1.43 (1.01-2.02) | 0.042 | 31440994 |
| Guo (2019) | Chinese | 1182 | rs145619195 | Overall | ANXA6 | 4q27 | C/T | Dominant | 0.65 (0.44-0.96) | 0.032 | 30536196 |
| Guo (2019) | Chinese | 1182 | rs117677079 | Overall | ANXA5 | 4q27 | C/T | Dominant | 1.65 (1.17-2.33) | 0.004 | 30536196 |
| Xu (2015) | Chinese | 510 | rs2071559 | Overall | VEGFR2 | 4q12 | C/T | Additive | 1.88 (1.43-3.01) | 0.018 | 26629211 |
| Bueno-Martínez (2022) | Spanish | 438 | rs1864183 | GBM | ATG10 | 5q14.2 | T/C | Recessive | 1.86 (1.15-3.01) | 0.018 | 35123435 |
| Xi (2022) | Chinese | 1094 | rs13177623 | Overall | CARMN | 5q32 | A/G | Dominant | 0.78 (0.61-0.99) | 0.043 | 35592549 |
| Guo (2020) | Chinese | 1031 | rs17057846 | Overall | MIR3142HG | 5q34 | A/G | Co-dominant | 1.93 (1.01-3.71) | 0.047 | 31513017 |
| Guo (2020) | Chinese | 1031 | rs2961920 | Overall | MIR3142HG | 5q34 | A/C | Additive | 1.20 (1.02-1.43) | 0.033 | 31513017 |
| Guo (2020) | Chinese | 1031 | rs58747524 | Overall | MIR3142HG | 5q34 | C/T | Additive | 1.22 (1.00-1.48) | 0.048 | 31513017 |
| Guo (2020) | Chinese | 1031 | rs7727115 | Overall | MIR3142HG | 5q34 | T/T | Dominant | 0.77 (0.61-0.98) | 0.033 | 31513017 |
| Li (2022) | Chinese | 1061 | rs62376564 | Overall | ARRDC3 | 5q14.3 | G/T | Recessive | 1.29 (1.04–1.60) | 0.021 | 34748117 |
| Pandith (2020) | Kashmiri | 316 | rs2736100 | Overall | hTERT | 5p15.33 | G/T | Additive | 3.00 (2.20-4.30) | < 0.001 | 32783258 |
| Pandith (2020) | Kashmiri | 316 | rs2736098 | Overall | hTERT | 5p15.33 | A/G | Additive | 1.70 (1.10-2.50) | 0.01 | 32783258 |
| Ding (2020) | Chinese | 1077 | rs2016520 | Overall | PPARD | 6p21.31 | C/T | Additive | 0.81 (0.67-0.99) | 0.037 | 32198386 |
| Ding (2020) | Chinese | 1077 | rs67056409 | Overall | PPARD | 6p21.31 | G/A | Additive | 0.82 (0.67-0.99) | 0.041 | 32198386 |
| Ding (2020) | Chinese | 1077 | rs1053049 | Overall | PPARD | 6p21.31 | C/T | Additive | 0.78 (0.64-0.95) | 0.012 | 32198386 |
| Yang (2022) | Chinese | 1061 | rs3808599 | Overall | NDRG1 | 8q24.22 | G/C | Recessive | 0.42 (0.19–0.90) | 0.025 | 33709284 |
| Yang (2022) | Chinese | 1061 | rs3802251 | Overall | NDRG1 | 8q24.22 | C/T | Additive | 0.79 (0.66–0.94) | 0.008 | 33709284 |
| Ding (2021) | Chinese | 1075 | rs13255292 | Overall | PVT1 | 8q24.21 | T/C | Recessive | 0.53 (0.29–0.99) | 0.046 | 33443959 |
| Huang (2020) | Chinese | 1051 | rs473426 | Overall | MAPKAP1 | 9q33.3 | C/G | Dominant | 1.53 (1.13-2.06) | 0.006 | 31773361 |
| Huang (2020) | Chinese | 1051 | rs1339499 | Overall | MAPKAP1 | 9q33.3 | C/T | Dominant | 0.74 (0.56-0.98) | 0.037 | 31773361 |
| Deng (2019) | Chinese | 1905 | rs2151280 | Overall | ANRIL | 9p21.3 | G/A | Dominant | 1.48 (1.21‐1.80) | 0.001 | 31489712 |
| Deng (2020) | Chinese | 1905 | rs3741219 | Overall | H19 | 11p15.5 | G/A | Additive | 0.54 (0.45–0.63) | < 0.001 | 32155588 |
| Zhang (2019) | Chinese | 1075 | rs7938889 | Overall | MAML2 | 11q21 | T/C | Recessive | 0.69 (0.50-0.95) | 0.023 | 31652449 |
| Zhang (2019) | Chinese | 1075 | rs485842 | Overall | MAML2 | 11q21 | T/C | Additive | 0.81 (0.67-0.98) | 0.032 | 31652449 |
| Guan (2021) | Chinese | 439 | rs7312175 | Overall | KRAS | 12p12.1 | A/G | Co-dominant | 1.66 (1.05-2.64) | 0.03 | 33850816 |
| Yang (2020) | Chinese | 1905 | rs11614913 | Overall | miR-196a-2 | 12q13.13 | T/C | recessive | 0.79 (0.64– 0.97) | 0.03 | 33230439 |
| Feng (2020) | Chinese | 1094 | rs7318578 | Overall | MIR17HG | 13q31.3 | C/A | Additive | 1.26 (1.07-1.49) | 0.006 | 33036577 |
| Bueno-Martínez (2022) | Spanish | 438 | rs3759601 | GBM | ATG2B | 14q32.2 | G/C | Dominant | 0.44 (0.27-0.74) | < 0.001 | 35123435 |
| Zhang (2019) | Chinese | 1075 | rs2239647 | Overall | AKAP6 | 14q12 | A/C | Recessive | 1.90 (1.19-3.03) | 0.007 | 31759389 |
| Pan (2013) | Chinese | 886 | rs861539 | Overall | XRCC3 | 14q32.33 | T/C | Additive | 1.17 (1.04-1.65) | < 0.05 | 23385236 |
| Mahjabeen (2021) | Pakistan | 470 | rs2169757 | Overall | CEP-152 | 15q21.1 | G/A | Additive | 6.92 (4.99–9.57) | < 0.001 | 34156311 |
| Bueno-Martínez (2022) | Spanish | 438 | rs2066844 | GBM | NOD2 | 16q12.1 | T/C | Dominant | 2.19 (1.24-3.88) | 0.006 | 35123435 |
| Zhang (2021) | Chinese | 1080 | rs2230742 | Overall | ADCY9 | 16p13.3 | A/G | Dominant | 0.63 (0.48-0.84) | 0.001 | 33326832 |
| Zhang (2021) | Chinese | 1080 | rs2531992 | Overall | ADCY9 | 16p13.3 | A/G | Dominant | 0.74 (0.56-0.99) | 0.041 | 33326832 |
| Al-Khatib SM (2020) | Arab Jordanian | 309 | rs799917 | GBM | BRCA1 | 17q21.31 | A/G | Recessive | 0.46 (0.26–0.82) | 0.01 | 32606887 |
| Yao (2019) | Chinese | 1905 | rs1059394 | Overall | TYMS | 18p11.32 | T/C | Dominant | 0.74 (0.55-0.99) | 0.04 | 31632074 |
| Zhou (2019) | Chinese | 1905 | rs1059394 | Overall | TYMS | 18p11.32 | T/C | Dominant | 0.74 (0.55–0.99) | 0.04 | 31525662 |
| Gao (2016) | Chinese | 495 | rs13181 | Overall | ERCC2 | 19q13.32 | G/T | Recessive | 1.87 (1.03-3.37) | 0.04 | 27323065 |
| Pan (2013) | Chinese | 886 | rs1799782 | Overall | XRCC1 | 19q13.31 | T/C | Additive | 1.76 (1.21–2.06) | ＜0.05 | 23385236 |
| Pan (2013) | Chinese | 886 | rs25487 | Overall | XRCC1 | 19q13.31 | A/G | Additive | 1.33 (1.02–1.64) | ＜0.05 | 23385236 |
| Yang (2020) | Chinese | 1905 | rs3746444 | Overall | miR-499-3p | 20q11.22 | G/A | Additive | 2.18 (1.82–2.60) | < 0.001 | 33230439 |
| Namgoong (2018) | Korean | 625 | rs6089953 | Overall | RTEL1 | 20q13.33 | G/A | Additive | 1.52 (1.17–1.97) | 0.001 | 30462709 |
| Namgoong (2018) | Korean | 625 | rs3848669 | Overall | RTEL1 | 20q13.33 | T/G | Additive | 1.54 (1.19–2.01) | 0.0009 | 30462709 |
| Namgoong (2018) | Korean | 625 | rs6010620 | Overall | RTEL1 | 20q13.33 | G/A | Additive | 1.55 (1.19–2.01) | 0.0009 | 30462709 |
| Namgoong (2018) | Korean | 625 | rs3787089 | Overall | RTEL1 | 20q13.33 | A/G | Additive | 1.55 (1.19–2.02) | 0.001 | 30462709 |
| Namgoong (2018) | Korean | 625 | rs6062302 | Overall | RTEL1 | 20q13.33 | C/T | Additive | 1.62 (1.26–2.10) | 0.0002 | 30462709 |
| Namgoong (2018) | Korean | 625 | rs115303435 | Overall | RTEL1 | 20q13.33 | A/G | Additive | 3.06 (1.69–5.54) | 0.0002 | 30462709 |

^a^ Allele, Effect allele/reference allele.

^b^ PubMed ID.
